# Incidence of catastrophic expenditures linked to obstetric and neonatal care at 92 facilities in Lubumbashi, Democratic Republic of the Congo, 2015

**DOI:** 10.1186/s12889-019-7260-9

**Published:** 2019-07-15

**Authors:** Abel Mukengeshayi Ntambue, Françoise Kaj Malonga, Karen D. Cowgill, Michèle Dramaix-Wilmet, Philippe Donnen

**Affiliations:** 1 Unité d’Epidémiologie et de Santé de la mère, du nouveau-né et de l’enfant, École de Santé Publique, Université de Lubumbashi, Lubumbashi, Democratic Republic of the Congo; 2 School of Interdisciplinary Arts and Sciences, University of Washington Tacoma, Tacoma, USA; 3 Department of Global Health, University of Washington, Seattle, USA; 4 Centre de recherche en Epidémiologie, Biostatistiques et recherche clinique, École de Santé Publique Université Libre de Bruxelles, Brussels, Belgium; 5 Centre de Recherche en Politiques et systèmes de santé-Santé internationale, École de Santé Publique Université Libre de Bruxelles, Brussels, Belgium

**Keywords:** Expenditures, health, Obstetric labor complications, Democratic Republic of the Congo

## Abstract

**Background:**

In the Democratic Republic of the Congo (DRC), more than 93% of users must pay out of pocket for care. Despite the risk of catastrophic expenditures (CE), 94% of births in Lubumbashi are attended by skilled personnel. We aimed to identify risk factors for CE associated with obstetric and neonatal care in this setting, to document coping mechanisms employed by households to pay the price of care, and to identify consequences of CE on households.

**Methods:**

We used mixed methods and conducted both a cross-sectional study and a phenomenological study of women who delivered at 92 health care facilities in all 11 health zones of Lubumbashi. In April and May 2015 we followed 1,627 women and collected data on their health care and household expenses to determine whether they experienced CE, defined as payments that reached or exceeded 40% of a household’s capacity to pay. Two months after discharge, we conducted semi-structured interviews with 58 women at their homes to assess the consequences of CE.

**Results:**

In all, 261 of 1,627 (16.0%) women experienced CE. Whether a woman or her infant experienced complications was an important contributor to her risk of CE; poverty, younger age, being unmarried, and delivering in a parastatal facility or with more highly trained personnel also increased risk. Among a subset of women with CE interviewed 2 months after discharge, those who were in debt or who had lost their trading income or goods were unable to pay their rent, their children’s school fees, or were obliged to reduce food consumption in the household; some had become victims of mistreatment such as verbal abuse, disputes with in-laws, denial of paternity, abandonment by partners, financial deprivation, even divorce.

**Conclusions:**

We found a higher proportion of CE than previously reported in the DRC or in other urban settings in Africa. We suggest that the government and funders in DRC support initiatives to put in place mutual-aid health risk pools and health insurance and introduce and institutionalize free maternal and infant care. We further suggest that the government ensure decent and regular payment of providers and improve the financing and functioning of health care facilities to improve the quality of care and alleviate the burden on users.

**Electronic supplementary material:**

The online version of this article (10.1186/s12889-019-7260-9) contains supplementary material, which is available to authorized users.

## Background

Skilled birth attendance is recognized as an intervention that reduces maternal and perinatal mortality [1]. The countries that have improved coverage in skilled birth attendance are also those that showed great progress toward attaining the target of Millennium Development Goal 5, to improve maternal health [2]. In Africa, skilled birth attendance has increased from 47% in 1990 to 78% in 2015 [3]. Now, Sustainable Development Goal 3.1–2 is focused on universal coverage of skilled birth attendance to attain a significant reduction in maternal and perinatal mortality by 2030 [1, 4]. Attaining this coverage has become a driving force of maternal health programs around the world [1, 4, 5].

However, this growth in the number of assisted deliveries brings with it an increase in the burden on the health system —in terms of infrastructure, medications, equipment, and human resources for health [1, 6]— the Every Mother, Every Newborn initiative [4] reaffirms the Abuja accords and insists on the responsibility of governments to allocate at least 15% of their budgets to health care, while encouraging new modes of financing for development in the post-2015 era.

In the Democratic Republic of the Congo (DRC), the budget allocated to health is low. In 2014, it was 10.8% of the total budget [7]. It corresponded to US$ 2–3 per resident per year, and represented only 30% of all total health expenditure. It was trivial compared to the minimum of US$ 86 per resident per year recommended in 2009 by the international high-level working group on health (members cited in [8]) and, even considering external contributions which comprise 40% of health system expenditures, the health expenditure per resident per year was still low (US$ 23) [7]. Given that nearly two thirds of this budget (63.9%) is taken up by health sector administration, the provision of health services has become entirely dependent on direct payments for care by users. More than 93% of users must pay to access care at health facilities [7].

In a country where the majority of the population is poor [9], basing financing for the functioning of health facilities on user fees entails two results [10]: first, care becomes inaccessible, especially for impoverished households; second, women who go to health facilities to deliver despite their poverty face a high risk of catastrophic expenses (CE) and reinforcement of their poverty. Expenses are defined as catastrophic when they are equal to or greater than 40% of the contributory capacity of a household [11]. CE is not always synonymous with a high cost of care; even relatively low expenditures can be disastrous for a poor household whose entire resources are needed for essentials. For these households, paying even a small sum can lead them into poverty [12].

For over a decade, initiatives aiming at universal coverage of health care have multiplied in the DRC [5, 13, 14]. In response to the Global Financing Facility (GFF) [14], the DRC has put in place a national policy of health care financing with the aim of increasing and harmonizing financing in support of national health plans and priorities, and of thus radically improving the results obtained in the area of women’s, infants’, and adolescents’ health [15]. At the national level in DRC, the incidence of CE linked to general care has been reported as 10.8% [15]. In 2014, in Lubumbashi, we showed that expenses tied to obstetric care were higher when compared to other cities in Africa [16], but information on the incidence of CE, its determinants and health and socioeconomic consequences among women who deliver with skilled attendance, is lacking. The rate of skilled birth attendance is high (94.5%) despite the extent of poverty in the city [17]; we wanted to discover the strategies used by households to meet these fees despite their impoverishing nature.

This study, which follows on one that described the variability of costs of obstetric and neonatal care in Lubumbashi, aims to identify risk factors for CE associated with obstetric and neonatal care in Lubumbashi [16]. We measured the incidence of CE and its determinants, documented coping mechanisms used by households to pay the price of care, and identified socioeconomic and health consequences of CE on households.

## Methods

### Study setting

The city of Lubumbashi covers an area of 747 km^2^ and has an estimated population of more than 2 million, for an average density of 2,543 inhabitants per km^2^ [18]; however, it comprises periurban areas where population density is lower. Nearly 70% of its population lives on less than US$1/day [9]. The city is divided into eleven Health Zones (HZ), all but one of which has a General Referral Hospital (GRH); on average, each HZ has 15 Health Centers (HC). More than 350 health care facilities (hospitals, polyclinics, and HCs) are found in Lubumbashi, with the majority – 70%— in the dense urban core [18]. The private sector accounts for more than 60% of facilities. Almost 180 facilities in Lubumbashi offer maternity services [19]. In 2012, 94.5% of deliveries were assisted [17].

### Population

We included facilities in all eleven HZs that had at least 25 deliveries in the month before our survey. In each of the 92 facilities that met the inclusion criteria, we invited all self-paying women admitted to the maternity to participate. To be included, women had to be able to speak French or another of the national languages, i.e., Swahili, Tshiluba, Lingala, or Kikongo to communicate with the researchers.

Women who could read and understand French were given the opportunity to read the written consent form themselves; for those who could not read or understand French, the form was read and explained by a researcher in another language in the presence of a person not involved in the study (e.g., a unit administrator) whose role was to confirm the content of the document. Women were given the opportunity to ask questions and then, if they chose, to sign the consent form. Information about each woman was kept confidential. All 1,627 women who gave birth in the selected health care facilities between April and May 2015 were eligible and gave consent to participate in the study. Inclusion criteria and selection methods for the qualitative portion that explored consequences of CE are described below, with the description of the semi-structured interview. This study was approved by the Medical Ethics Committee of the University of Lubumbashi (UNILU/CEM/010/2011).

### Study

We used mixed methods and conducted both a cross-sectional study and a phenomenological study [20]. This allowed us to appreciate two complementary aspects of CE tied to obstetric and neonatal care. The cross-sectional study (quantitative) allowed us to investigate the determinants of CE, while phenomenology permitted us to determine the short- to mid-term consequences of these costs that could not have been adequately captured by the quantitative approach. For the latter, we made home visits 2 months after participants’ discharge from the maternity unit. Interviews and document reviews were carried out by thirty trained assistants.

### Data collection

#### Structured interview and document review

We used a structured questionnaire (Additional file 1) to capture information about the expenditures —including the reason for each payment— related to delivery, as well as sociodemographic and economic information not included in the medical record. From each woman’s medical record, we collected data on reasons for admission, presence of complications, type of delivery and we collected documentation of expenditures linked to delivery. We consulted the head of the maternity unit to determine what charges were covered by each payment.

We followed payments made daily for the entire length of stay [10], apart from receipts available from the women or their family members, or in the files of the maternity unit if the bill was not available. Payments were validated after triangulation of information obtained i) from the woman giving birth or her family, ii) in the medical record, and iii) by confirmation from the head of the maternity unit. For medications purchased outside the facility or from staff at the facility, we documented payments based on receipts or, if the receipt was missing, based on the woman’s report, but only if the medication was identified, or the information validated by the birth attendants. This was also the case for laboratory tests and blood purchased at the blood bank. If the total declared by the woman was higher than that reported by the maternity team, and the proof of expenditures were not available, the difference was attributed to the category “other fees”, which often included gratuities given to the health care personnel.

#### Semi-structured interviews

Semi-structured individual interviews (Additional file 2) were conducted only at homes. We drew up an a priori list addressing three essential themes: i) the woman’s view on the quality of care she received; ii) her view of the price of care at the maternity unit; and iii) the consequences (problems) she faced as a result of the payments made at the maternity. Considering each facility in which a woman delivered as a “cluster”, the saturation threshold was fixed at four redundant interviews in a cluster that didn’t add anything to advancing the conceptualization of the consequences of CE [20]. In the end, we interviewed 58 women in 10 clusters. The interviews were recorded, then transcribed.

### Data management and analysis

The data from the structured interviews and document review were double-entered and stored in Excel (Additional file 3). The total costs of care at delivery was the sum of expenditures tied to obstetric and neonatal care (medications, supplies, the act of delivery, cesarean section, episiotomy, dressings, lab tests, newborn care, stay, and medical record) [10].

To determine the household’s capacity to pay, we estimated total expenditures —comprising payments for food and other (housing, electricity, water, school fees, holidays, birthdays, trips, clothing, furniture, communication, transport, leisure, fund transfers, as well as the costs of delivery)– for the year preceding the survey [12]. We asked women to report recurring expenditures like food, communication, transportation, and leisure for the month preceding the survey, and used these to estimate for the entire year; for other non-food expenditures, we estimated them for the whole year [21]. We subtracted the food expenditures to obtain the household’s capacity to pay. We defined CE for obstetric and neonatal care equal to or greater than 40% of the household’s capacity to pay [12, 22, 23]. All expenditures were expressed in US$ (at the time of this study, 920 Congolese francs (CDF) = US$1).

To evaluate the impact of socioeconomic level on the incidence of CE, we created a socioeconomic index [24]. We used principal components analysis (PCA) based on the variables used in urban settings in the 2013 Demographic and Health Survey (DHS) [25]: household (number of people per household, relationship with the owner of the house, materials of the wall and roof, source of water and electricity, source of energy for the household, type of toilets), presence of radio, telephone, freezer, television, computer, fan, sewing machine, wheeled vehicle (bicycle, motorcycle, car), type of bed and chairs. The socioeconomic well-being score was derived from the first principal component, which was used to construct the household socioeconomic index [25]. Apart from the number of people per household, variables were dichotomized [25]. The percent of variance explained by the first component was 36.1% for this model.

We described the profile of health care facilities and of participants included in the study with standard descriptive statistics (proportion, mean and standard deviation, median, minimum, and maximum). We used Mann-Whitney and Kruskal-Wallis tests to compare expenditures for each type of delivery between women who suffered CE and those who did not to test the hypothesis of differences in expenditures between these two groups. Bonferroni’s correction was used for two-by-two comparisons of expenditures by type of delivery (uncomplicated vaginal, complicated vaginal and cesarean delivery) and the occurrence of CE. We used a chi-squared test, with a significance threshold of 5%, to compare the incidence of CE (dependent variable) by characteristics of women, socioeconomic level (low vs high), presence of a more vs less highly skilled birth attendant, private ownership of the facility (vs public or religious), the occurrence of obstetric or neonatal complications (vs uncomplicated delivery), and a long stay in the maternity unit (vs a stay of 3 days or fewer) (independent variables). In the logistic regression model, all these variables were adjusted by the demographic characteristics of the women. We calculated relative risk (RR) and 95% confidence interval (CI) to evaluate the strength of the association between the variables listed above and the incidence of CE.

We used forward stepwise logistic regression with a significance threshold of 5% to identify determinants of CE, with resulting adjusted odds ratios (aOR) and 95% CI. We included only independent variables with a *p*-value of ≤ 0.10 in univariable analysis in the multivariable analysis. To avoid collinearity between obstetric and neonatal complications and related care, we built two models, one based on health care and the other on obstetric and neonatal complications. We tested the fit of the final model with the Hosmer-Lemeshow test [26]. All analyses were conducted with Stata v13.1. We defined complicated delivery according to WHO criteria as “medical problems associated with obstetric labor, such as hemorrhage (antepartum and postpartum), obstructed labor, postpartum sepsis, placenta praevia, placental abruption and premature rupture of membranes, complications of abortion, severe pre-eclampsia and eclampsia, ectopic pregnancy and ruptured uterus or others”. Neonatal care was categorized into three levels: basic care offered to healthy newborns, emergency neonatal care (EmNC) for newborns with intrapartum respiratory distress, and intensive care for sick or low-birth-weight newborns [27].

Data from the semi-structured interviews were simultaneously translated from local languages into French at the time of their transcription, then re-read. Initially, we analyzed them to pull out the major consequences, then we conducted a thematic analysis about the i) economic, ii) social, and iii) health consequences of CE and the possible associations among them [20].

## Results

### Profile of women

We included 1,627 women in our study. Table 1 shows that in the majority (61.1%) of women were recruited in the HZs of Ruashi (21.8%), Lubumbashi (16.8%), Kampemba (11.4%), and Katuba (11.1%). The HZs of Kisanga (8.2%), Vangu (7.8%), Mumbunda (7.7%), Tshamilemba (6.3%), Kenya (5.0%), and Kamalondo (3.8%) made up the remaining 38.9%. Over the course of the survey, there were no deliveries at the HC in the Kowe HZ. The majority of women were recruited in HCs (42.0%) and GRHs (35.3%). Polyclinics represented 13.5% of women, while the tertiary hospitals, Sendwe and University Clinics of Lubumbashi (UCL), each accounted for only 4.6% of women. More than half the women (864, or 53.2%) came from private-sector facilities; one quarter of these came from faith-based facilities; the public sector accounted for 40.3% of participants, and parastatal health facilities for 6.6%.

**Table 1:** Profile of parturient women surveyed, Lubumbashi, DRC, 2015

Variables	Total (*n* = 1627)	Percent
Health Zones		
Lubumbashi	274	16.8
Katuba	181	11.1
Mumbunda	126	7.7
Vangu	127	7.8
Kisanga	133	8.2
Ruashi	354	21.8
Kamalondo	61	3.7
Tshiamilemba	103	6.3
Kenya	82	5.0
Kampemba	186	11.4
Type of facility		
Health center	684	42.0
Sendwe (provincial referral hospital)	74	4.6
General referral hospital	574	35.3
University Clinics of Lubumbashi (UCL)	75	4.6
Polyclinics	220	13.5
Sector of ownership		
Private, religious	214	13.1
Public	655	40.3
Para-statal commercial	108	6.6
Private, non-religious	650	40.0
Age (years)		
< 20	172	10.6
20–34	1215	74.6
≥ 35	240	14.8
Marital status		
Married	1589	97.7
Not married	38	2.3
Partner’s age (years)		
< 25	114	7.0
25–34	784	48.2
35–44	589	36.2
≥ 45	115	7.1
Unknown	25	1.5
Wealth quintile		
Q1 (very poor)	302	18.6
Q2	317	19.5
Q3	311	19.1
Q4	344	21.1
Q5 (very rich)	353	21.7

The average age of women in the sample was 27 years (minimum 13, maximum 47). The average age of the partners of those who were married was 34 years (min 17, max 64). Thirty-eight women (2.3%) were not married. We divided participating women into quintiles by wealth.

More than a third (35.8%) of women arrived at the maternity unit on foot or by motorcycle or bicycle, 39% arrived by public transport, and only a quarter arrived (25.2%) by private transport. One hundred fourteen (7.0%) of surveyed women had been referred from another health care facility. In all, 659 women (40.5%) had at least one complication during or following delivery. Obstructed labor (14.8%), cervical and perineal tears (13.2%), ante- and post-partum hemorrhage (5.3%), and post-partum infections (3.1%) were the most commonly reported complications. One hundred ninety-seven women (12.1%) delivered by caesarean. All the newborns born to women who had complications stayed in the neonatal intensive care unit, 24.9% to receive emergency neonatal care and 74.1% to receive intensive care for ill newborns.

At delivery, 88.9% of women were attended by midwives and 6.0% by generalist physicians, while 5.2% were attended by specialist physicians. The median length of stay in the maternity unit was 3 days (min 1, max 64). More than three quarters (79.1%) of women had pre-selected the maternity unit where they would deliver. Nearly half of all women (48.4%) delivered without knowing in advance how much they would pay. In all, 261 of the 1,627 women recruited for the study (16.0%; 95% CI 14.3–17.9%) reported catastrophic expenses related to obstetric and neonatal care.

In all, 261 of 1,627 (16.0%) women experienced CE. In Table 2, the median expenditure for delivery was higher in women who experienced CE (US$ 54) compared with those who did not (US$ 38; *p* < 0.001). In looking at each type of delivery, we noted that the median expenditures for a complicated vaginal birth were not significantly different between women who experienced CE and those who did not (*p* = 0.69). On the other hand, the median expenditure for those with uncomplicated vaginal births who experienced CE was US$4 higher than for those who did not experience CE, and those with cesarean sections and CE spent US$65 more than those who did not experience CE (*p* = 0.043) or caesareans (*p* = 0.005).

**Table 2:** Median total expenditures and total capacity to pay by type of delivery and experience of catastrophic expenses, city of Lubumbashi, DRC, 2015 (Mann-Whitney test with Bonferroni correction)

Type of delivery	Non-catastrophic expenses		Catastrophic expenses		*p*
	Total (n)	Median in US$ (minimum-maximum)	Total (n)	Median in US$ (minimum-maximum)	
Total expenditures for obstetric and neonatal care					
Uncomplicated vaginal	906	34 (10–220)	95	38 (10–163)	0.043
Complicated vaginal	356	43 (12–396)	73	42 (12–272)	0.69
Caesarean	104	323 (82–966)	93	388 (108–924)	0.005
Total	1366	38 (10–966)	261	54 (10–924)	< 0.001*
Total household capacity to pay					
Uncomplicated vaginal	906	104 (20–2462)	95	65 (20–222)	0.03
Complicated vaginal	356	122 (19–3009)	73	54 (20–199)	0.04
Caesarean	104	569 (112–3201)	93	392 (55–1297)	0.031
Total	1366	114 (19–3201)	261	123 (20–1297)	0.015*

*Kruskal-Wallis

Furthermore, we noted that the median household capacity to pay (contributive capacity) was US$9 lower among women who had CE compared to those who did not (*p* = 0.015) regardless of the type of delivery.

In Table 3, the incidence of CE was different by HZ. Women who delivered in the HZ of Kisanga, Ruashi, Kamalondo,Tshiamilemba, Kenya, and Kampemba incurred CE less than half as often as those who delivered in the HZ of Lubumbashi. The incidence of CE ranged from 18.3 to 32.6% in the HZs of Lubumbashi, Mumbunda, and Katuba, but the differences did not attain statistical significance. The incidence likewise varied by type of facility. The incidence of CE was almost two times higher in the provincial hospital (Sendwe) and in the GRH compared to the Health Centers. Women who delivered in facilities that were public, private non-religious, or owned by a parastatal entity had more than two times the risk of having CE compared to those who delivered in private religious facilities.

**Table 3:** Incidence of catastrophic expenses by HZ, type and sector of health care facility, Lubumbashi, DRC, 2015

Variables	Total	Incidence of CE (%)	RR	95% CI	*p*
Health Zones					< 0.001
Lubumbashi	274	25.9	1		
Katuba	181	32.6	1.3	0.9–1.7	
Mumbunda	126	18.3	0.7	0.5–1.1	
Vangu	127	17.3	0.7	0.4–1.0	
Kisanga	133	15.0	0.6	0.4–0.9	
Ruashi	354	11.6	0.5	0.3–0.6	
Kamalondo	61	8.2	0.3	0.1–0.7	
Tshiamilemba	103	7.8	0.3	0.2–0.6	
Kenya	82	4.9	0.2	0.1–0.5	
Kampemba	186	4.3	0.2	0.1–0.3	
Type of facility					< 0.001
Health center	684	11.6	1		
Sendwe (provincial referral hospital)	74	23.0	2.0	1.2–3.1	
General referral hospital	574	20.0	1.7	1.3–2.3	
University Clinics of Lubumbashi (UCL)	75	18.7	1.6	0.9–2.6	
Polyclinics	220	16.4	1.4	0.9–2.0	
Sector of ownership					< 0.001
Private, religious^a^	214	6.1	1		
Public	655	20.5	3.4	2.0–5.8	
Para-statal commercial	108	12.0	2.0	1.1–4.1	
Private, non-religious	650	15.5	2.6	1.5–4.5	

^a^Used for comparison because these are the health facilities in which the price of care is most stable and where care is perceived by women to be of good quality

In Table 4, younger age of the woman, although not of her partner, was associated with the variation in incidence of CE. Unmarried women had more than twice the risk of having CE compared to those who were married. The incidence of CE was 16 and 23 times higher, respectively, among the lowest and second-lowest wealth quintiles compared to the highest.

**Table 4:** Incidence of catastrophic expenses (CE) by sociodemographic and economic characteristics of women, evolution of the delivery and length of stay in the maternity unit, Lubumbashi, DRC, 2015

Variables	Total	Incidence of CE (%)	RR	95% CI	*p*
Age (years)					0.10
< 20	172	20.4	1.6	0.9–2.5	
20–34	1215	16.1	1.3	0.9–1.9	
≥ 35	240	12.5	1		
Marital status					< 0.001
Married	1589	15.5	1		
Not married	38	36.8	2.4	1.5–3.5	
Partner’s age (years)					0.39
< 25	114	14.9	0.8	0.4–1.4	
25–34	784	16.7	0.9	0.6–1.3	
35–44	589	15.3	0.8	0.5–1.2	
≥ 45	115	19.1	1		
Unknown	25	4.0	0.2	0.1–1.1	
Wealth quintile					< 0.001^*^
Q1 (very poor)	302	44.7	22.5	11.0–46.8	
Q2	317	31.6	15.9	7.7–33.3	
Q3	311	3.2	1.6	0.7–4.1	
Q4	344	2.6	1.3	0.5–3.4	
Q5 (very rich)	353	2.0	1		
Referral status					< 0.001
Referred	114	40.4	2.8	2.2–3.6	
Not referred	1513	14.2	1		
Complications					< 0.001
Post-partum infection	50	58.0	9.1	6.4–12.5	
Pre & post-partum hemorrhage	86	41.9	6.5	4.6–9.1	
Eclampsia	27	40.7	6.4	3.6–10.1	
Placenta Prævia	22	31.8	5.0	2.5–8.8	
Soft-tissue tears	214	25.7	4.0	2.9–5.6	
Placental abruption	20	25.0	3.9	1.7–7.7	
Obstructed labor	240	23.3	3.6	2.6–5.1	
None	968	6.4	1		
Type of delivery					< 0.001
Uncomplicated vaginal	1001	9.5	1		
Complicated vaginal	429	17.0	1.8	1.4–2.4	
Caesarean	197	47.2	5.0	3.9–6.3	
Neonatal care					< 0.001
Basic	971	9.1	1		
Emergency	163	39.9	4.4	3.3–5.8	
Intensive	493	21.9	2.4	1.9–3.1	
Type of skilled birth attendant					< 0.001
Nurses and midwives	1446	14.0	1		
General physician	97	29.9	2.1	1.5–2.9	
Specialist physician	84	35.7	2.6	1.8–3.4	
Delivery facility					0.03
Planned	1287	15.0	1		
Not planned	340	20.0	1.3	1.1–1.7	
Length of stay (days)					< 0.001
≤ 3	1143	12.4	1		
4–14	419	19.8	1.6	1.2–2.0	
≥ 15	65	55.4	4.5	3.4–5.7	

*Chi-squared test for trend

Table 4 also shows that women who were referred from one facility to another had nearly three times the risk of CE compared to those who were not. Women who had any complication —eclampsia, placenta praevia, obstructed labor, pre- or post-partum hemorrhage, or uterine rupture— had at least three and a half times higher risk of CE compared with those who had no complications.

Women who had complicated vaginal births had nearly twice the risk of CE compared with those who had an uncomplicated birth, and those who delivered by caesarean had five times the risk compared to those who had an uncomplicated delivery.

Among women whose newborns received emergency or intensive care, the incidence of CE was 2.4–4.4 times higher compared to mothers of healthy newborns. It was also more than two times higher when deliveries were managed by generalist or specialist physicians as opposed to nurses and midwives.

Women who did not plan to give birth in the facility in which they ended up giving birth had nearly twice the risk of CE as compared to those who delivered in the planned facility. The length of stay increased the risk of CE. Women who stayed more than 3 days in the maternity unit had at least twice the risk of CE compared with those who stayed no more than 3 days.

The multivariable model (Table 5) shows results similar to those seen in the univariable analyses. Poverty, management of obstetric or neonatal complications, provision of care by highly qualified personnel, care provided in facilities of parastatal entities and referral in the course of delivery – all factors associated with higher charges – increased the risk of CE.

**Table 5:** Determinants of catastrophic expenses among parturient women surveyed, Lubumbashi, DRC, 2015

Variables	[*n* = 1627; CE = 261]		
	aOR	95% CI	*p*
Sector^a&b^			0.65
Public vs private religious	1.3	0.6–2.6	
Parastatal entity vs private religious	1.3	0.5–3.8	
Private non-religious vs private religious	1.5	0.7–3.2	
Not married vs married^a&b^	1.6	0.7–3.4	0.24
Socioeconomic level^a&b^			< 0.001
Q1 vs Q5	38.7	17.6–85.2	
Q2 vs Q5	23.5	10.7–51.8	
Q3 vs Q5	1.6	0.6–4.2	
Q4 vs Q5	1.3	0.5–3.6	
Referred vs not referred^a^	1.9	1.1–3.6	< 0.001
Type of delivery^a^			< 0.001
Complicated vaginal vs uncomplicated	1.9	1.2–2.9	
Caesarean vs uncomplicated	7.5	3.6–15.4	
Obstetric complications^b^			< 0.001
Infections vs none	20.8	7.4–58.0	
Pre & post-partum hemorrhage vs none	9.4	5.0–17.7	
Eclampsia vs none	4.7	1.5–15.0	
Placenta Prævia vs none	5.6	1.5–21.0	
Soft-tissue tears vs none	4.2	2.5–7.1	
Placental abruption vs none	2.4	1.2–9.5	
Obstructed labor vs none	4.8	2.8–8.2	
Newborn care^a^			< 0.001
Emergency vs basic	2.8	1.4–5.4	
Intensive vs basic	2.9	1.9–4.3	
Skilled birth attendant at delivery^a^			< 0.001
Generalist physician vs midwives	2.2	0.9–5.1	
Specialist physician vs midwives	3.9	1.8–8.5	
Length of stay (days)^b^			< 0.001
3–14	1.3	0.9–1.9	
≥ 15	3.6	1.5–8.1	

^a^included only in model 1, built on the basis of care received by women and newborns; ^b^included only in model 2, built on the basis of complications requiring obstetric and neonatal care; ^a & b^included in both models

### Alternatives for the payment of obstetric and neonatal care

#### Quantitative aspects

**Fig. 1:**
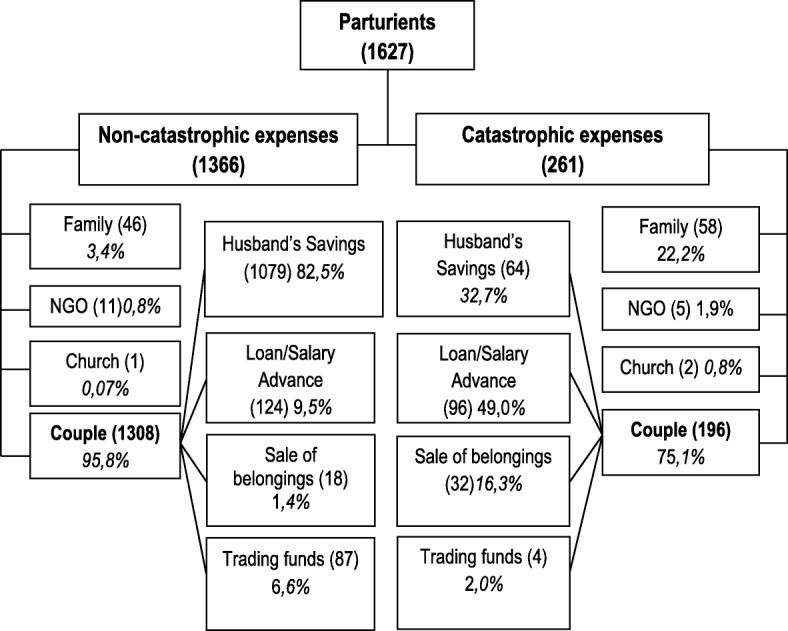
Modes and payment options for charges for care for households

The means of payment of maternity fees differed for women who suffered CE and those who did not. In Fig. 1, we see that, even though in both groups the woman and her partner were the main source for payment of the maternity fees, the proportion of women assisted by non-governmental organizations (NGO) and family was at least twice as high among women with CE as among those without CE. Among the couples who had paid the maternity fees themselves, the origin of fees paid were different between the two groups (*p* < 0.001). The proportion of those who were able to draw on savings was higher among women who were not victims of CE (82.5%) than among those who were (32.7%). Similarly, the proportion of women who used their trading capital (funds that enabled the woman or her partner to engage in commercial activities) to pay the charges for care was three times higher among women who were not victims of CE (6.6%) than among those who were (2.0%). The proportion of couples who had borrowed or requested a salary advance and that of couples who had sold or mortgaged household goods to pay the charges for care was higher among women who had experienced CE as opposed to those who had not.

#### Qualitative aspects

Even though women who suffered CE were able to draw on various options to pay fees, they perceived the cost of care – unknown at admission – as a great burden which the whole family had to struggle to confront. Following are selected statements by participants who experienced CE:

*“I didn’t know it was a caesarean, I didn’t even know how much I had to pay; I was referred urgently from a health center.” [Participant 1]*

*“I didn’t know how much it should cost, the nurses were discussing with my sister.” [Participant 2]*

*“I planned to save in the last month of my pregnancy, but the birth was premature, it took us by surprise.” [Participant 3]*

*“Not a single medication was given to us by the maternity [unit]; we always paid for them out of our own pocket [woman or family] … if we’d had the money for the hospital stay and the surgical intervention, I would have left immediately, because I was not ill.” [Participant 4]*

As shown above in the quantitative part, families resorted to different options to finance health care fees.

*“At the time of the last caesarean, my family paid for everything even though they shouldn’t have; this time, he (husband) had to get by with his family.” [Participant 5]*

*“My husband gave me everything, I don’t know how he did it, I don’t know how much he earns approximately per month; I think at least he did what was necessary to save during the pregnancy.” [Participant 6]*

*“My husband hasn’t worked for several months, and I didn’t work either in the last months of my pregnancy: I had no money when I delivered, I didn’t know who would pay these fees; but my family and my in-laws did.” [Participant 7]*

*“In any case, my husband is a man: he had to manage, this time my family should not have to take on so much expense.” [Participant 8]*

### Consequences to households of catastrophic expenses associated with obstetric and neonatal care

When women experience CE for obstetric and neonatal care at health facilities, it changes their standard of living and households must confront difficult choices: “treat the woman and the newborn and let the family die of hunger, or not”. This study allowed us to observe that there are many consequences of these expenditures.

#### Economic Consequences

In terms of economics, women who had CE were first of all insolvent. They were indebted and used their trading funds or sold their goods to pay these bills. Because of their insolvency, these women were detained in the maternity unit and denied care such as bandages, analgesics, and newborn cord care.

*“Since I was in the hospital, I couldn’t trade and I couldn’t help my husband: everything was screwed [messed up]. On his own, he had to pay for everything: food, school fees, transportation, clothes, etc. In these conditions, that’s how we didn’t have money to pay for health care.” [Participant 9]*

*“I had to stop my petty trading, to stay in the hospital until someone pays all the bills.” [Participant 10]*

*“As for the children, I have seven, and they stay by themselves at home because their dad is a motorcyclist [motorcycle taxi driver] and he stays downtown late to earn what’s needed; my oldest daughter even got pregnant.” [Participant 11]*

*“I had asked the nurses to keep my baby if they wanted, and to let me go look for money until I could pull together the necessary sum.” [Participant 12]*

*“We pawned some of our clothes, the chairs, our flatscreen and my capital (trading money), with a view to getting the first installment of the fees … when the hospital asked us to pay the rest, we took on debt from one of our neighbors.” [Participant 13]*

#### Quality of care during the stay in the maternity

They were also victims of disrespectful care.

*“There were days when I didn’t receive paracetamol and my dressing wasn’t changed because I hadn’t yet paid even a part of the fees for treatment … my baby’s cord was also not cleaned because I hadn’t paid for their medications.” [Participant 14]*

*“Because I was slow to pay for care, the person who took care of me wouldn’t greet me in the morning; she didn’t ask me how I’d slept, either.” [Participant 15]*

*“Before I’d paid for the care, when the midwife changed my dressing, she didn’t speak to me. When she spoke to me, it was to remind me that I should pay the bill. I couldn’t even complain about the pain when she rubbed my wound … I was afraid she would stop the dressing since I hadn’t yet paid.” [Participant 16]*

#### Household subsistence

After leaving the maternity, women who were in debt or those who had lost their trading income or goods were unable to pay their rent, their children’s school fees, or were obliged to reduce food consumption in the household.

*“Since I got out of the maternity, we haven’t paid our rent. The landlord threw us out. And since we weren’t able to find food with the children, I came here to my parents’ house until I can resume my activities, if I find money … the friends we borrowed from for the maternity come every day demanding payment and threatening to take us to court.” [Participant 17]*

*“My two children don’t go to school because we haven’t paid the fees for 3 months … they are with my parents, because here sometimes we go to sleep hungry.” [Participant 18]*

#### Relationships with partner and family

Women have been victims of marital conflict and mistreatment – verbal abuse, threats of divorce, disputes with in-laws, denial of paternity, absconding of partner, financial deprivation, or dissolution of the partnership—since they are viewed as the source of excessive expenses. The following statements shed light on some of the consequences on households of catastrophic expenditures tied to obstetric and neonatal care.

*“Since the delivery, my husband has left the house on the pretext that he was going to look for money … his friends and family have told me that I made him carry the responsibility for the pregnancy.” [Participant 19]*

*“We hadn’t quarreled, but he didn’t come see me at the hospital, not a single member of his family came to see me; they didn’t even think that I also needed to eat, it was my aunt who always brought me food.” [Participant 20]*

*“My mother-in-law said I wasn’t fit because I delivered by caesarean.” [Participant 21]*

*“They (my in-laws) told me that I was the cause of the unhappiness or curse of their child [my husband] … because of me, everything he does doesn’t work … and that my pregnancy made him poor.” [Participant 22]*

#### Access to care for other members of the household

CE has likewise been at root of inability to financially access other care (postnatal and preschool consultations, consultations for illness) for the woman and other members of the household. This has led them to resort to other means of management (self-medication, traditional care, or prescriptions in a drugstore).

*“Since I had no money to go to the health center, when my daughter fell ill, I went to get, on credit, malaria medications from the pharmacy of my friend’s little brother.” [Participant 23]*

*“After discharge from the maternity, I didn’t go back to the hospital for the weighing and vaccination of the child: I didn’t have the fees for transport and to pay to the hospital … I’m using traditional medicine for hemorrhoids which bother me since the delivery.” [Participant 24]*

**Fig. 2:**
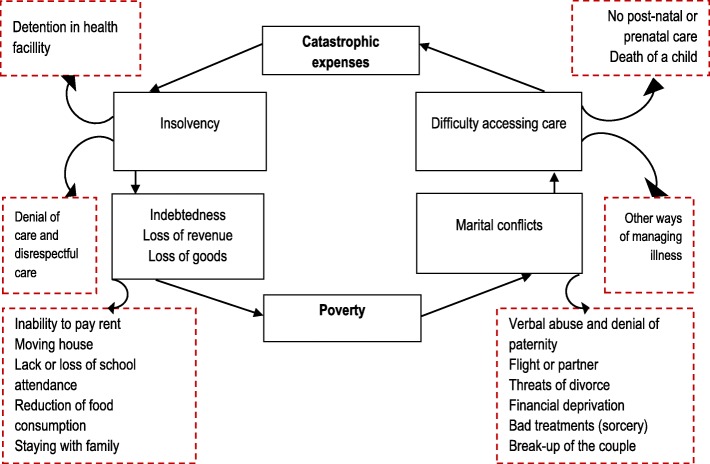
Short-term consequences of catastrophic expenses (CE) linked to obstetric and neonatal care in the city of Lubumbashi, DRC, 2015. (points framed in black: direct consequences of CE; points framed in dashed red lines: indirect consequences of CE; black arrows: relationships among consequences of CE)

In Fig. 2, we show a schematic of the consequences tied to CE from obstetric and neonatal care in Lubumbashi.

## Discussion

In this study, we found that 16.0% of women were victims of CE linked to obstetric and neonatal care. This incidence was higher if the women were poor, had maternal or neonatal complications, or were treated by highly trained health care personnel. This is higher than the overall incidence of catastrophic expenses due to health care reported in 2014 in DRC by the World Bank, which was 10.8% [15]. In an earlier study [16], we showed that, given that the transportation network in Lubumbashi is easily accessible and affordable, the contribution of the price of transport to the total payment at the time of delivery remains low (3.2–8.2%). Thus, even without taking transport into account, the incidence of CE in Lubumbashi is higher than in urban settings in various sub-Saharan African countries [28–31].

In our study, we found that women in the two lowest wealth quintiles had, respectively, 23 and 16 times higher risk of CE than those in the highest. In several cities of Africa, similar results have been reported [28–32]. It is not surprising that without health insurance or universal coverage, the poor should be at higher risk of CE. The dependence of the health care facilities and health care system on user payments has weakened the leadership of the state and its regulatory power over price-setting for care so that, in a city where more than 60% of the facilities are private [19] and where neither mutual aid organizations or health insurance exists [18], it is cost of living for health care providers, rather than rational factors linked to the functioning of the facility, that determines the variability in the price of care. Each facility has its prices for care, and within a facility, for the same acts, women may pay different amounts. This is seen even in public facilities presumed to function for the public good [7]. Further, 5% of the revenue of these facilities is paid to the central office of the HZ and so on, up to the Ministry of Health [11].

Complications and the extra fees they entail also increase risk of CE. We noted that when there were complications at delivery, women had at least twice the risk of CE. Medical and nursing services, the amount of medications used, newborn care, the length of stay, and complementary care in the case of referrals are, in general, responsible for this increase.

High-quality obstetric care certainly has costs, whether these are supported by users or by other players [33]. When health expenditures become catastrophic, they constitute a barrier to the use of care, limit its quality, and reinforce the poverty of households [10]. Insufficient salaries from the state lead health care providers to impose additional fees for each service [34]. To improve their income, the GRHs and HCs overlap the services they offer in their care bundles [35]. When primary care that would normally be provided at the HC level is provided in the GRHs or polyclinics, it costs at least twice as much. Women pay different amounts for the same services according to whether they are cared for by a midwife, a generalist physician, or a specialist physician. HCs are over-medicalized, offering services that ought to be offered only at higher levels of the health care system. This contributes to the increase in price of primary care [35], and to a lack of continuity of care within the system; women who are referred have to pay more than those who are not. Furthermore, in addition to paying for services, women must buy medications, supplies, and equipment, either from providers or from the pharmacy in the hospital or from outside the hospital. Because this is a sector in which the state has little control [11], the risk of overbilling is very high, given that these things constitute a source of extra income for the facilities and the staff.

Most women who encounter these exorbitant charges are not prepared for them, because they don’t anticipate having complications. The consequences they face are major, ranging from detention in the maternity unit in the short term, to sliding further into poverty in the medium term. Several studies [10, 28, 36] report similar observations and also note that these consequences are likely to persist beyond the short to the medium, and long term in the life of the household.

### Mechanisms for coping with direct out-of-pocket payments

Why, in a city where 70% of the population is poor [9] and care financially unaffordable [7], does the rate of deliveries attended by skilled personnel remain high? Mobilization of clan in the form of contributions from close and extended family members to pay for pregnancy –and delivery– related health care not only attenuates economic consequences but also justifies subsequent health care provider-assisted deliveries despite these families’ poverty. But this clan solidarity has consequences. Because they are considered the cause of excessive expenditures, women whose health care costs reach catastrophic levels because of complications may become victims of mistreatment such as verbal abuse, disputes with in-laws, denial of paternity, abandonment by partners, financial deprivation, even divorce – all of which are factors likely to reinforce their poverty [10].

### Alternatives for health care financing in the DRC

Initiatives that aim to alleviate the price of obstetric care – either through result-based or health systems strengthening approaches– have been tested in several countries in Africa [37–46]. De Brouwere et al. [47] report that results are mixed. According to these authors, “the schemes studied in the context of financing obstetric care have not shown, in an absolute way, either growth in use of services or improvement of results in terms of health. In terms of equity, there was little support for reduction of CE” [47] (our translation).

Witter et al. [48] document the elements to take into account to implement a policy to protect women from high price of care. These elements corroborate the determinants of CE we observed in the city of Lubumbashi. They write: “adopting a good package of measures for a given context cannot be done in a mechanical fashion. The balance between the constraints of supply and demand may vary, and the conception of an appropriate policy should consider the availability of resources, the cultural expectations of roles and responsibilities, as well as the way in which the health services are financed and organized” (our translation). Emphasizing the determinants of CE in the city of Lubumbashi, our study shows that approaches aiming to protect women from CE should not be one-dimensional. On the demand side, there is the socioeconomic level of the population; on the supply side, there is the organizational context, the quality of care, available financing, management of human resources for health, and cost of living.

Dimensions like these can only be taken into account in the context of a broad strategy of financing and health systems strengthening. This was the case for Morocco [49] and Rwanda [50], which obtained remarkable results in terms of maternal health after considerable reforms and a large increase in the financing of the health care system. This has not been the case in the DRC because the National Plan for Health Development (2010–2015) has not been fully implemented. According to the United Nations Millennium Project 2005 [51], “practical investments and policies for a functioning health system include training and retaining competent, motivated health workers, strengthening management systems, providing adequate supplies of essential drugs, and building clinics and laboratory facilities. Eliminating user fees for essential health services, improving community health education, promoting behavior change, and involving communities in decision-making and service delivery are also critical measures”.

For example, in the current context of organization and provision of obstetric and neonatal care in Lubumbashi, it is urgently necessary to reinforce the leadership of the Ministry of Health to oversee and regulate the establishment and functioning of health care facilities that provide obstetric and neonatal care. The ministry should also oversee and regulate respect for the technical platform and continuity of care between the different levels of the health care system, as well as the standardization of fees according to the type of care provided to mothers and infants.

### Study limitations

In this setting, patients don’t always request bills or invoices for expenses; this carries with it a risk of bias, since women may have forgotten an expenditure made by a family member [21], while staff may have intentionally avoided mentioning informal payments like gratuities [10]. Such a bias could have contributed to an underestimation of payments by the woman. To significantly reduce the impact of this bias, we triangulated the data collected from various sources, as described in the methods. It is also possible that the food expenses of households were underestimated given that purchases are very fragmented over the course of the month [21], which increases the risk of forgetting some during our interviews. Such a bias would also have caused an underestimate of the incidence of CE.

## Conclusion

In this study, we measured the incidence of CE, its determinants, coping mechanisms used by households to pay the price of care, and identified socioeconomic and health consequences of CE.

We observed that direct payment for obstetric and neonatal care causes CE. The women who are vulnerable to CE are those with a low socioeconomic status and who have obstetric or neonatal complications. Also, the dependence on the health care system, including payments to staff, on user fees increases the risk of these expenditures. CE causes a cycle of poverty and constitutes a barrier to universal coverage of respectful, high-quality basic and emergency obstetric and neonatal care.

To guarantee universal coverage of high-quality health care, we suggest that the government and funders in DRC support initiatives to put in place mutual-aid health risk pools and health insurance, which are still rare in the DRC, and that they introduce and institutionalize free maternal and infant care.

Also, by guaranteeing a decent and regular payment of providers and improving the financing and functioning of health care facilities, the government could substantially reduce the portion of system expenditures carried by households via direct payment as a condition for access to care.

## Additional files

Additional file 1: Expenses for obstetric and neonatal care in Lubumbashi: data collectiontool. (DOC 349 kb)

Additional file 2: Interview guide. (DOCX 15 kb)

Additional file 3: Description of data. (XLSX 596 kb)

## Data Availability

All data generated or analyzed during this study are included in this published article and its supplementary information files.

## Abbreviations

95%CI: 95% confidence interval
aOR: Adjusted Odds Ratio
CDF: Congolese Francs
CE: Catastrophic expenditure
DHS: Demographic and Health Survey
DRC: Democratic Republic of the Congo
EmNC: Emergency Neonatal Care
GFF: Global Financing Facility
GRH: General Reference Hospital
HC: Health Centre
HZ: Health Zone
NGO: Non-governmental organizations
PCA: Principal component analysis
Q: Quintile
RR: Relative Risk
UCL: University Clinics of Lubumbashi
UN: United Nations
US$: US Dollar
WHO: World Health Organization
