# Supplementary material for: Incidence of catastrophic expenditures linked to obstetric and neonatal care at 92 facilities in Lubumbashi, Democratic Republic of the Congo, 2015
Source: BMC Public Health. 2019 Jul 15;19:948. doi: 10.1186/s12889-019-7260-9 (PMC6632186; doi:10.1186/s12889-019-7260-9)
Supplement: Supplementary file 2 — Interview guide. (DOCX 15 kb) [file 12889_2019_7260_MOESM2_ESM.docx]

**INTERVIEW GUIDE**

1. Could you tell us, when you were pregnant, how did you prepare for your delivery, in terms of:
   1. *Location of the health facility where you should give birth,*
   2. *The means of transport to use,*
   3. *The person to contact in case of complications;*
   4. *Fees to be paid for care in the health facility.*
2. How did you appreciate the fees that the health facility required?
   1. *Compared to the obstetric and neonatal care you received in the health facility;*
   2. *Compared to the fees you expected to pay*
3. To the extent that you have considered the fees for obstetric and neonatal care as expensive and beyond your expectations and means, can you explain how you managed to pay for it?
   1. *Explain the alternatives used to pay the fees*
   2. *Explain the additional period of detention before paying these fees*
4. Can you explain to us the consequences that these excessive costs of obstetric and neonatal care have had on:
   1. *Your stay in the maternity ward*
   2. *The quality of your relationship with health care staff*
   3. *The quality of your health care and that of your baby*
5. Can you explain to us, after leaving the maternity ward, the consequences that these excessive costs of obstetric and neonatal care have had on:
   1. *Your own life*
   2. *That of your baby*
   3. *The life of your household*
   4. *Relationship with your spouse*
   5. *Relationships with your own family*
   6. *And relationships with your in-laws*
6. Do you think what could be the links between these different consequences?
